# Treating Cutaneous T-Cell Lymphoma with Highly Irregular Surfaces with Photon Irradiation Using Rice as Tissue Compensator

**DOI:** 10.3389/fonc.2015.00049

**Published:** 2015-02-24

**Authors:** Lonika Majithia, Yi Rong, Farzan Siddiqui, Todd Hattie, Nilendu Gupta, Michael Weldon, Arnab Chakravarti, Henry K. Wong, Pierluigi Porcu, Meng Xu-Welliver

**Affiliations:** ^1^Department of Radiation Oncology, James Cancer Hospital, The Ohio State University, Columbus, OH, USA; ^2^Department of Radiation Oncology, University of California Davis Comprehensive Cancer Center, Sacramento, CA, USA; ^3^Department of Radiation Oncology, Henry Ford Health System, Detroit, MI, USA; ^4^Division of Dermatology, Department of Internal Medicine, James Cancer Hospital, The Ohio State University, Columbus, OH, USA; ^5^Division of Hematology, Department of Internal Medicine, James Cancer Hospital, The Ohio State University, Columbus, OH, USA

**Keywords:** cutaneous T-cell lymphoma, radiotherapy, tissue compensation, irregular surface

## Abstract

**Purpose:** Cutaneous T-cell lymphoma (CTCL) is known to have an excellent response to radiotherapy, an important treatment modality for this disease. In patients with extremity and digit involvement, the irregular surface and depth variations create difficulty in delivering a homogenous dose using electrons. We sought to evaluate photon irradiation with rice packing as tissue equivalence and determine clinical tolerance and response.

**Materials and methods:** Three consecutive CTCL patients with extensive lower extremity involvement including the digits were treated using external beam photon therapy with rice packing for tissue compensation. The entire foot was treated to 30–40 Gy in 2–3 Gy per fraction using 6 MV photons prescribed to the mid-plane of an indexed box filled with rice in which the foot was placed. Treatment tolerance and response were monitored with clinical evaluation.

**Results:** All patients tolerated the treatment without treatment breaks. Toxicities included grade 3 erythema and desquamation with resolution within 4 weeks. No late toxicities were observed. All patients had a partial response by 4 weeks after therapy with two patients achieving a complete response. Patients reported improved functionality after treatment. No local recurrence has been observed.

**Conclusion:** Tissue compensation with rice packing offers a convenient, inexpensive, and reproducible method for the treatment of CTCL with highly irregular surfaces.

## Introduction

Cutaneous T-cell lymphomas (CTCL) are a rare subset of primary extra-nodal non-Hodgkin’s lymphomas of the skin that derive from mature T-cells, with peak incidence in the 55–60 years age range. The most common histological subtypes of CTCL are mycosis fungoides (MF), Sezary syndrome (SS), and CD30+ lymphoproliferative disorders, such as anaplastic large cell lymphoma (ALCL) and lymphomatoid papulosis (LyP). Rare types include adult T-cell lymphoma (ATL), extra-nodal NK/T-cell lymphoma (ENKTL), and panniculitis-like T-cell lymphoma (SPTCL) (1–3). CTCL are generally indolent lymphoid neoplasms that present with recurring symptomatic skin lesions (plaques, patches, tumors) for which multiple treatment modalities have been beneficial. For MF, skin-directed therapies, with or without the addition of systemic therapy, represent an important component of the overall management plan across all stages and histological subtypes of CTCL. Superficial skin-directed therapy options include topical steroids, phototherapy, photodynamic therapy, and radiotherapy (4–6). Systemic therapy options include biologic therapies, immuno-modulators, and chemotherapy (5, 7).

Cutaneous T-cell lymphomas are exquisitely sensitive to radiotherapy. Ionizing radiation induces cell death predominantly by apoptosis in hematopoietic lineages, and is able to achieve complete response (CR) at a much lower dose compared to solid cancers. Radiotherapy is known to palliate symptoms and improve local disease control in cutaneous lymphomas (8–10). Previously published studies demonstrate that there is a dose–response relationship, which include a CR of lesions to doses over 2000 cGy for fractionated regimens (11) and 700 cGy for single-fraction regimens (12, 13). Various types of radiotherapy have been utilized for skin irradiation such as kilo-voltage photons (superficial/orthovoltage), electrons, and mega-voltage photons with tissue compensation. Electron beam therapy is advantageous as it reduces deep tissue radiation penetration and reduces toxicity to visceral organs. For CTCL, electron beam therapy is most commonly used in the palliative setting, when one or several isolated cutaneous lesions are treated for symptom control (14, 15). Less commonly, when there is extensive skin involvement, total skin electron beam irradiation is employed (16).

However, for regions with highly irregular surfaces, such as the feet with digit involvement, electron field setup can prove challenging with inadequate tumor coverage and excess dose variance. Photon irradiation with tissue compensation can be utilized here. Conventional tissue compensation, such as water baths, increases the risk of infection with prior skin wounds. Here, we describe methods and preliminary outcome data of photon irradiation with rice packing in three patients with CTCL and extensive involvement of the entire foot including digits as an alternative to electron treatment to achieve improved dose homogeneity.

## Materials and Methods

Between January 2012 and March 2013, three patients presented with CTCL involving the lower extremity and the digits. Two patients had advanced MF while one patient had localized ALCL. One patient had bilateral extremity involvement and two patients had single extremity involvement. Patient suffered from extremity pain, swelling, inability to ambulate, wound infections, and pruritus. Patient data and medical histories are provided in Table 1. Palliative radiotherapy was recommended for symptom palliation and local disease control.

**Table 1:** **Patient data and medical histories**.

Patient	Age/gender	Diagnosis	Diagnosis date	Prior treatments	Extremity involvement	RT dose	Concurrent CT
A	70 years old M	Advanced stage MF	7/2007	Topical steroids, RT, CT, phototherapy, biologic	Bilateral	30 Gy in 2 Gy/fx	None
B	54 years old M	Stage IB MF	5/2004	Biologic, RT, topical steroids, CT	Left	30 Gy in 3 Gy/fx	Romidepsin
C	74 years old M	Stage IE ALCL	2010	Surgical resection, CT	Left	40 Gy in 2 Gy/fx	MTX

*MF, mycosis fungoides; ALCL, anaplastic large cell lymphoma; RT, radiotherapy; CT, chemotherapy; Gy/fx, Gray per fraction; MTX, methotrexate*.

**Figure 1:**
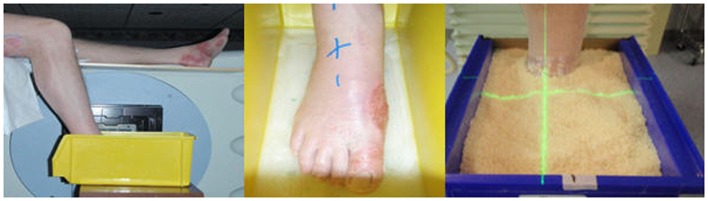
**Clinical setup for patient A**.

**Figure 2:**
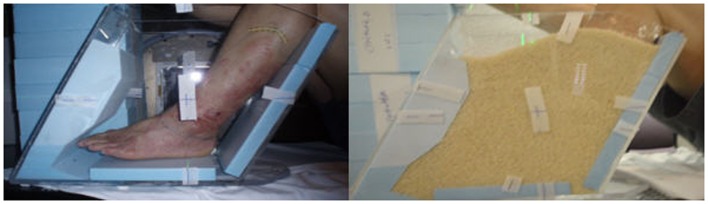
**Clinical setup for patient B**.

All three patients were treated using rice as packing material. This reduced the risk of open wound infections, provided immobilization of extremities, and improved homogeneity in dose delivery. Though direct comparison was not made with other materials, the reduced infection risk and ease of use with rice packing were preferred. Institutional review board (IRB) approval was obtained before patient treatment and data analysis. The density of the rice packing was evaluated based on the computer tomography scan HU value and was equal to that of water (average HU = 0 with range of ± 100 HU). A plastic tank with dimensions of 18″ × 8″ × 9″ was utilized with non-slip base for patient A (Figure 1). After noting difficulty in measuring lateral quantities in colored plastic and with the angle of the patient’s leg, a custom container was created and used for the remainder of patients. This new container used transparent plastic slabs sealed together for ease in clinical setup and a slanted posterior edge to allow the leg to rest easily (Figure 2). The container provided sufficient room for the involved extremity and adequate water-equivalent buildup. The foot was wrapped in a clear wrap with plantar surface flat against base. After the foot was positioned, the container was filled with rice to the superior border of the treatment field. All other patients were setup in a similar fashion.

**Figure 3:**
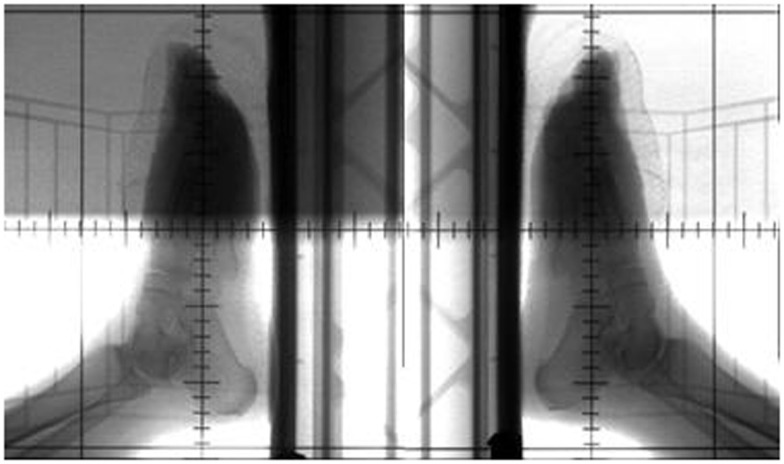
**Acuity simulation films for patient C**.

**Figure 4:**
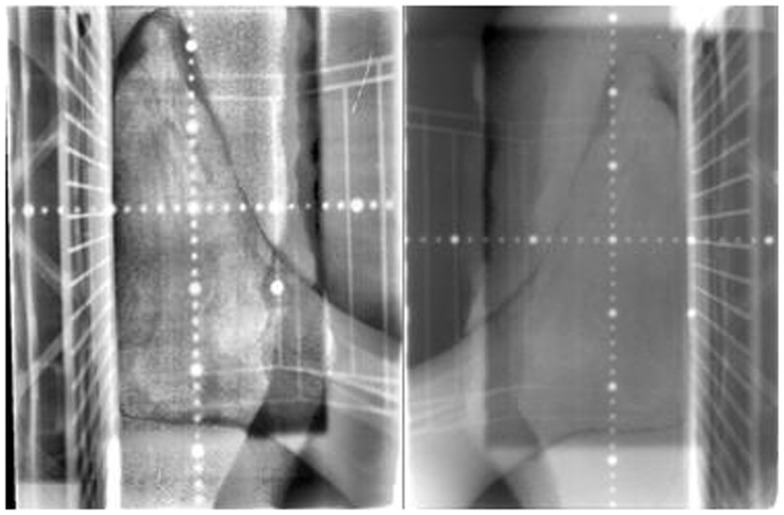
**MV portal films of left lateral (left) and right lateral (right) for patient C**.

Treatment plans were manually created to deliver two lateral radiation fields at 90° and 270° gantry angles. Kilo-voltage simulation images were taken using the Acuity™(Varian Medical Systems, Palo Alto, CA, USA) system prior to treatment for the determination of treatment field size and blocks by the physician (Figure 3). Isocenter was determined on the Acuity system and markings were made on the skin of patient, as well as on the plastic tank. MUs were manually calculated based on the prescription dose, setup distance, and field size, without considering heterogeneity correction. As shown in Figure 4, 2 MV portal images were taken for each patient from the two lateral opposed fields utilizing 6 MV photon energies. A maximum field size of 40 cm × 40 cm was determined for both gantry angles, with MLCs to shape the beam. Adequate beam flashing was given circumferentially around the foot, to allow large setup uncertainties and dose buildup. All patients were positioned supine with the leg flexed. Special arrangements, including vacuum bags and knee cushions, were used to keep the other leg out of the treatment field. During the course of treatment, weekly portal films were taken for treatment verification. As shown in Figure 4, bony anatomy was verified on the portal films with reference to the reticule. Dose was prescribed to the mid-plane of the plastic tank. Dose prescriptions were 30–40 Gy in 2–3 Gy fractions. Radiation was delivered using a Siemens MXE Mevatron linear accelerator. Three optically stimulated luminescence dosimeters (OSLDs) were placed at the lateral and dorsal aspects of the foot to measure dose directly at skin surface of all patients. With adequate water-equivalent buildup, the only setup uncertainty that was not accounted for was the lateral positioning (i.e., the foot can be off-centered and closer to one beam but further away from the other). But with the two-field arrangement at both lateral gantry angles, dose deviation was considered minimal with the lateral positioning offset. Based on standard radiation practice, the setup was considered reproducible daily for consecutive treatments. The National Cancer Institute Common Terminology Criteria for Adverse Events (CTCAE) version 3.0 was used to determine the toxicity scale. The response to radiotherapy was evaluated clinically with photographs obtained at each follow-up after 1, 3, 6, and 12 months. The response to therapy has recently been established in “Clinical End Points and Response Criteria in MF and Sezary Syndrome” using data from multiple international lymphoma organizations (17). Since we are only discussing local therapy in this study, in addition to the CR, partial response (PR), stable disease (SD), and progressive disease (PD) described by the consensus guidelines, we further define “complete local response” as no clinical evidence of disease or wound in the treatment field and “partial local response” as >50% reduction of lesion size in the treatment field.

## Results

**Figure 5:**
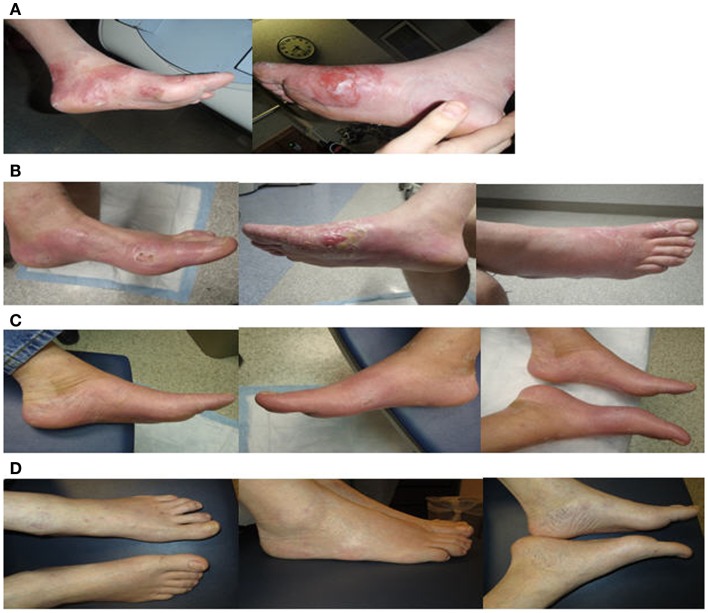
**A series of clinical photographs of patient A before treatment (A), immediately after treatment (B), 2 months after treatment (C), and 12 months after treatment (D)**.

**Figure 6:**
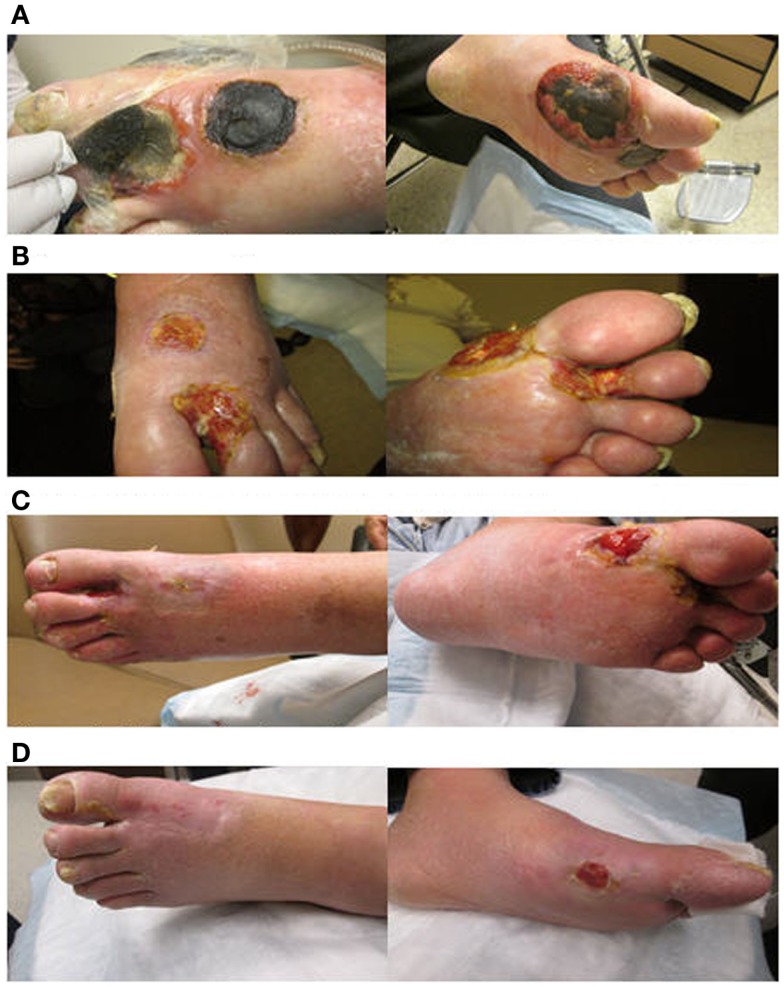
**A series of clinical photographs of patient C before treatment (A), 2 months after treatment (B), 6 months after treatment (C), and 12 months after treatment (D)**.

Dosimetric parameters were first measured. OSLD measurements at lateral plantar surface revealed an average dose of 221 cGy, or 110.5% of prescription dose, for patient A, suggesting adequate dose buildup at the skin. Overall, a variation of the radiation dose received by the skin did not exceed 2–11% of the prescribed dose due to the use of rice packing in all patients. The setup was reproducible daily for 10–20 consecutive treatments verified by light field confirmation daily and by portal films weekly. All patients noticed resolution of initial symptoms by 1–3 months after therapy. All three patients showed a PR by the end of their treatment, as seen in Figure 5 for patients A and Figure 6 for patient C. Two patients had complete local response 4 weeks post-treatment, and the third patient had partial local response after 12 months. One patient returned for their 20-month assessment and remained disease free at bilateral feet.

Treatment toxicities included grade 3 erythema, desquamation, pain, and temporary difficulty with ambulation due to extremity swelling. Acute symptoms subsided by 4 weeks. The skin remained smooth and well-vascularized without residual radiation toxicities at 6–12 months, as shown in Figure 5 for patient A and Figure 6 for patient C. With a median follow-up of 14 months (range 9–20 months), there have been no local recurrences within the boundaries of the radiation field. One patient passed away <22 months after treatment completion from progressive systemic disease. The two other patients remain on systemic therapy.

## Discussion

The role of radiotherapy has been well established in cutaneous B-cell and T-cell lymphoma for palliative symptom management and local disease control. The majority of published studies review the role of electron therapy focusing on adequate dose deposition at the skin surface and a reduced deep tissue toxicity (4, 18, 19). The dose–response relationship in CTCL has been well documented. In one retrospective review, PR was observed with a single dose of 100 cGy, but CRs required dosages greater than or equal to 700 cGy (4, 12). In several retrospective reviews, a local control rate of 75% at 5 years post-therapy in stage I MF has been reported. Authors used conventionally fractionated radiotherapy with 1.8–2 Gy per fraction to a total dose of 20–40 Gy (10). The role of hypofractionated therapy has been tested by Neelis et al. among 49 patients (126 disease sites) with cutaneous B-cell lymphoma and MF who were treated in two fractions of 2–4 Gy per fractions (20). Seventy percent of patients with MF failed to respond to 4 Gy delivered in two fractions but only 8% failed to respond when 8 Gy in two fractions were given, suggesting that higher dose is needed for this group of patients. In a disease with systemic involvement, systemic therapies are used with some success.

Disease site coverage with electron radiotherapy may be difficult due to the presence of air gaps and tissue interfaces such as in extremities and digits. Electron radiotherapy may be used, but dose homogeneity may be achieved easier with photon radiotherapy using a tissue mimetic. In our study, we have used rice packing as a tissue equivalent compensator to achieve a homogenous dose distribution and dose buildup at the skin surface. Dosimetric data done preliminarily in our clinic has demonstrated the equivalence of rice packing and water in electron density. Rice packing offers a reproducible and relatively simple clinical setup for patients with cutaneous lymphoma involving complex surface areas, such as extremities. The responses observed in our patients are representative of previously reported data with 100% PR and 75% CR in the treatment fields. Toxicities are equivalent to those seen traditionally with electron therapy, including erythema desquamation, and pain. No significant late toxicities have been observed with a maximum 20-month follow-up. Drawbacks of photon irradiation are that there is no sparing of deep tissues and the extremity skin surface is treated circumferentially. Therefore, this approach is not suited for tumors requiring higher doses. However, this treatment appears to be safe in the dose range for cutaneous lymphoma. Future directions of research include the evaluation of safety and activity of this approach in larger patient populations and utility of radio-sensitizing agents concurrently with photon therapy for extremity irradiation for cutaneous lymphomas and other disease sites.

## Conclusion

Photon irradiation with tissue compensation with rice packing offers a novel, inexpensive, and reproducible method for treating CTCL of the extremities when highly irregular surface is involved. This is a practical method and the outcomes observed in our patients have been excellent.

## Conflict of Interest Statement

The authors declare that the research was conducted in the absence of any commercial or financial relationships that could be construed as a potential conflict of interest.

## References

[1] Wong HK Mishra A Hake T Porcu P. Evolving insights in the pathogenesis and therapy of cutaneous T-cell lymphoma (mycosis fungoides and Sezary syndrome). Br J Haematol (2011) 155:150–66. 10.1111/j.1365-2141.2011.08852.x 21883142 PMC4309373

[2] Willemze R Jaffe ES Burg G Cerroni L Berti E Swerdlow SH WHO-EORTC classification for cutaneous lymphomas. Blood (2005) 105:3768–85 10.1182/blood-2004-09-3502 15692063

[3] Bradford PT Devesa SS Anderson WF Toro JR. Cutaneous lymphoma incidence patterns in the United States: a population-based study of 3884 cases. Blood (2009) 113:5064–73. 10.1182/blood-2008-10-184168 19279331 PMC2686177

[4] Hoppe RT. Mycosis fungoides: radiation therapy. Dermatol Ther (2003) 16:347–54. 10.1111/j.1396-0296.2003.01647.x 14686978

[5] Wollina U Cutaneous T cell lymphoma: update on treatment. Int J Dermatol (2012) 51:1019–36 10.1111/j.1365-4632.2011.05337.x 22909354

[6] Knobler E. Current management strategies for cutaneous T-cell lymphoma. Clin Dermatol (2004) 22:197–208. 10.1016/j.clindermatol.2003.12.003 15262305

[7] Trautinger F Knobler R Willemze R Peris K Stadler R Laroche L EORTC consensus recommendations for the treatment of mycosis fungoides/Sezary syndrome. Eur J Cancer (2006) 42:1014–30. 10.1016/j.ejca.2006.01.025 16574401

[8] De Sanctis V Osti MF Berardi F Ardito F Valeriani M Martelli M Primary cutaneous lymphoma: local control and survival in patients treated with radiotherapy. Anticancer Res (2007) 27:601–5. 17348448

[9] Micaily B Moser C Vonderheid EC Koprowski C Lightfoot D Markoe A The radiation therapy of early stage cutaneous T-cell lymphoma. Int J Radiat Oncol Biol Phys (1990) 18:1333–9. 10.1016/0360-3016(90)90306-5 1695214

[10] Wilson LD Kacinski BM Jones GW. Local superficial radiotherapy in the management of minimal stage IA cutaneous T-cell lymphoma (mycosis fungoides). Int J Radiat Oncol Biol Phys (1998) 40:109–15. 10.1016/S0360-3016(97)00553-1 9422565

[11] Cotter GW Baglan RJ Wasserman TH Mill W. Palliative radiation treatment of cutaneous mycosis fungoides – a dose response. Int J Radiat Oncol Biol Phys (1983) 9:1477–80. 10.1016/0360-3016(83)90321-8 6195138

[12] Kim JH Nisce LZ D’Anglo GJ. Dose-time fractionation study in patients with mycosis fungoides and lymphoma cutis. Radiology (1976) 119:439–42. 10.1148/119.2.439 772750

[13] Thomas TO Agrawal P Guitart J Rosen ST Rademaker AW Querfeld C Outcome of patients treated with a single-fraction dose of palliative radiation for cutaneous T-cell lymphoma. Int J Radiat Oncol Biol Phys (2013) 85:747–53. 10.1016/j.ijrobp.2012.05.034 22818412

[14] Micaily B Miyamoto C Kantor G Lessin S Rook A Brady L Radiotherapy for unilesional mycosis fungoides. Int J Radiat Oncol Biol Phys (1998) 42:361–4 10.1016/S0360-3016(98)00218-1 9788416

[15] Muche JM Gellrich S Sterry W Treatment of cutaneous T-cell lymphomas. Semin Cutan Med Surg (2000) 19:142–8 10.1016/S1085-5629(00)80012-7 10892717

[16] Maingon P Truc G Dalac S Barillot I Lambert D Petrella T Radiotherapy of advanced mycosis fungoides: indications and results of total skin electron beam and photon beam irradiation. Radiother Oncol (2000) 54:73–8. 10.1016/S0167-8140(99)00162-0 10719702

[17] Olsen EA Whittaker S Kim YH Duvic M Prince HM Lessin SR Clinical end points and response criteria in mycosis fungoides and Sezary syndrome: a consensus statement of the international society for cutaneous lymphomas, the United States cutaneous lymphoma consortium, and the cutaneous lymphoma task force of the European organisation for research and treatment of cancer. J Clin Oncol (2011) 29:2598–607. 10.1200/JCO.2010.32.0630 21576639 PMC3422534

[18] Akilov OE Grant C Frye R Bates S Piekarz R Geskin LJ. Low-dose electron beam radiation and romidepsin therapy for symptomatic cutaneous T-cell lymphoma lesions. Br J Dermatol (2012) 167:194–7. 10.1111/j.1365-2133.2012.10905.x 22372971 PMC3386371

[19] Hoppe RT Fuks Z Bagshaw MA. Radiation therapy in the management of cutaneous T-cell lymphomas. Cancer Treat Rep (1979) 63:625–32. 87276

[20] Neelis KJ Schimmel EC Vermeer MH Senff NJ Willemze R Noordijk EM. Low-dose palliative radiotherapy for cutaneous B- and T-cell lymphomas. Int J Radiat Oncol Biol Phys (2009) 74:154–8. 10.1016/j.ijrobp.2008.06.1918 18834672

